# Diagnostic Prediction of portal vein thrombosis in chronic cirrhosis patients using data-driven precision medicine model

**DOI:** 10.1093/bib/bbad478

**Published:** 2024-01-13

**Authors:** Ying Li, Jing Gao, Xubin Zheng, Guole Nie, Jican Qin, Haiping Wang, Tao He, Åsa Wheelock, Chuan-Xing Li, Lixin Cheng, Xun Li

**Affiliations:** The First Hospital of Lanzhou University, Lanzhou, China; Respiratory Medicine Unit, Department of Medicine & Centre for Molecular Medicine, Karolinska Institutet, Stockholm, Sweden; Heart and Lung Centre, Department of Pulmonary Medicine, University of Helsinki and Helsinki University Hospital, Helsinki, Finland; The First School of Clinical Medicine, Lanzhou University, Lanzhou, China; School of Computing and Information Technology, Great Bay University, Guangdong, China; The First Hospital of Lanzhou University, Lanzhou, China; School of Computing and Information Technology, Great Bay University, Guangdong, China; The First Hospital of Lanzhou University, Lanzhou, China; Jilin Hepato-Biliary Diseases Hospital, Changchun, China; Respiratory Medicine Unit, Department of Medicine & Centre for Molecular Medicine, Karolinska Institutet, Stockholm, Sweden; Department of Respiratory Medicine and Allergy, Karolinska University Hospital, Stockholm, Sweden; Respiratory Medicine Unit, Department of Medicine & Centre for Molecular Medicine, Karolinska Institutet, Stockholm, Sweden; Shenzhen People's Hospital, The First Affiliated Hospital of Southern University of Science and Technology, The Second Clinical Medical College of Jinan University, Shenzhen, China; The First Hospital of Lanzhou University, Lanzhou, China

**Keywords:** portal vein thrombosis, chronic cirrhosis patients, diagnostic prediction, data-driven precision medicine model, machine learning

## Abstract

**Background:**

Portal vein thrombosis (PVT) is a significant issue in cirrhotic patients, necessitating early detection. This study aims to develop a data-driven predictive model for PVT diagnosis in chronic hepatitis liver cirrhosis patients.

**Methods:**

We employed data from a total of 816 chronic cirrhosis patients with PVT, divided into the Lanzhou cohort (*n* = 468) for training and the Jilin cohort (*n* = 348) for validation. This dataset encompassed a wide range of variables, including general characteristics, blood parameters, ultrasonography findings and cirrhosis grading. To build our predictive model, we employed a sophisticated stacking approach, which included Support Vector Machine (SVM), Naïve Bayes and Quadratic Discriminant Analysis (QDA).

**Results:**

In the Lanzhou cohort, SVM and Naïve Bayes classifiers effectively classified PVT cases from non-PVT cases, among the top features of which seven were shared: Portal Velocity (PV), Prothrombin Time (PT), Portal Vein Diameter (PVD), Prothrombin Time Activity (PTA), Activated Partial Thromboplastin Time (APTT), age and Child–Pugh score (CPS). The QDA model, trained based on the seven shared features on the Lanzhou cohort and validated on the Jilin cohort, demonstrated significant differentiation between PVT and non-PVT cases (AUROC = 0.73 and AUROC = 0.86, respectively). Subsequently, comparative analysis showed that our QDA model outperformed several other machine learning methods.

**Conclusion:**

Our study presents a comprehensive data-driven model for PVT diagnosis in cirrhotic patients, enhancing clinical decision-making. The SVM–Naïve Bayes–QDA model offers a precise approach to managing PVT in this population.

## INTRODUCTION

Portal vein thrombosis (PVT) is characterized by the intraluminal occurrence of thrombosis within the portal vein, encompassing its left and right hepatic branches and potentially extending into the splenic vein and superior mesenteric vein. This leads to consequential complete or partial obstruction of the portal vein’s blood flow, representing a clinically significant vascular complication that is particularly prominent among individuals afflicted with liver cirrhosis [1]. In this context, non-malignant PVT manifests in approximately 25% of cirrhotic patients, presenting significant challenges to prognosis and clinical management [2]. The clinical consequences of PVT are extensive, encompassing mortality, hemorrhage, ascites, acute kidney injury and post-liver transplantation, emphasizing the critical importance of early detection and intervention, particularly in the setting of liver cirrhosis [3, 4]. Research has indicated that risk factors such as the widening of the portal vein diameter, deceleration of portal vein velocity, poor liver function and other variables contribute to the development of liver cirrhosis and PVT [5, 6]. Despite this, the precise cause and mechanism of PVT remain elusive. Consequently, regular monitoring and early detection of PVT are crucial. Timely implementation of active anticoagulation therapy has demonstrated a significant improvement in prognosis and outcomes for affected individuals.

Currently, we still lack an ideal diagnostic method for PVT that is repeatable, easily accessible and secure and imposes minimal financial or psychological burdens on patients. To date, imaging continues to be a central component in diagnostic methodologies for PVT. Initial screening and diagnosis are often facilitated through ultrasound, prized for its non-invasiveness and relatively high accuracy [7]. However, the interpretative reliability of ultrasound findings can be influenced by a constellation of factors, including the patient’s physiological status, potential vascular anomalies and the expertise of the ultrasonographer. To achieve definitive diagnosis, computed tomography (CT) and magnetic resonance imaging (MRI) are deployed, each with its unique attributes and limitations [8, 9]. CT, while effective, is encumbered by concerns surrounding radiation exposure and potential nephrotoxicity linked to contrast agent administration [8]. Conversely, MRI, owing to its radiation-free profile and heightened sensitivity, presents an appealing alternative, albeit tempered by considerations of cost and quality variability [9]. Complementary to imaging, hematology and coagulation blood tests stand as fundamental diagnostic pillars, providing indispensable insights into PVT risk assessment and diagnosis [10]. These tests encompass a gamut of parameters, such as hemoglobin, platelet count, prothrombin time, activated partial thromboplastin time and D-dimer levels, as well as liver and renal function markers [11, 12].

While the diagnostic methods discussed are indeed pivotal, the intricate interplay of diverse clinical and laboratory factors underscores the necessity for a comprehensive data-driven precision medicine model. Presently, the majority of prediction models in PVT among chronic cirrhosis patients are built on single-center data. This approach results in diverse clinical outcomes due to variations in patient genetics and living environments throughout the country. Machine learning, a multidisciplinary field, can enhance clinical prediction models by leveraging its ability to simulate human learning behavior, continuously refining knowledge structures for improved performance. This model is crucial in achieving a higher degree of precision and individualization when predicting the risk of PVT in patients grappling with chronic cirrhosis. Early diagnosis of cirrhotic PVT holds paramount importance. Yet, it is notable that the landscape lacks multicenter-based studies equipped to accurately predict the risk of developing cirrhotic PVT. In this context, the development of such a model becomes not only advantageous but also indispensable. Within the dynamic landscape of modern medical science, data-driven precision medicine models have been applied in disease diagnosis and outcome predictions, such as individualized pair analysis (iPAGE) [13, 14, 15, 16, 17], least absolute shrinkage and selection operator (LASSO) [18, 19, 20] and deep neural networks [21, 22]. As precision medicine is catalyzing profound transformations in healthcare paradigms, our study takes on a mission of paramount significance. We embark on the journey to bridge a critical diagnostic gap by harnessing advanced modeling techniques, including Support Vector Machine (SVM), Naïve Bayes, Quadratic Discriminant Analysis (QDA) and SHapley Additive exPlanations (SHAP). These models are meticulously integrated into a sophisticated data-driven precision medicine framework poised to revolutionize our approach to diagnosing and managing PVT in the context of chronic cirrhosis. We further trained the model with multicenter data to achieve higher accuracy. We further trained the model with multicenter data to achieve higher accuracy.

Our overarching objective is to reshape diagnostic precision, enabling more effective clinical decision-making. Through this pioneering effort, we aim to fulfill an urgent need in the management of this multifaceted medical condition. The diagnostic prediction of PVT in chronic cirrhosis patients using a data-driven precision medicine model stands as a transformative endeavor with the potential to significantly enhance patient care and optimize clinical outcomes.

## MATERIALS AND METHODS

### Patient selection from two clinical cohorts

**Figure 1:**
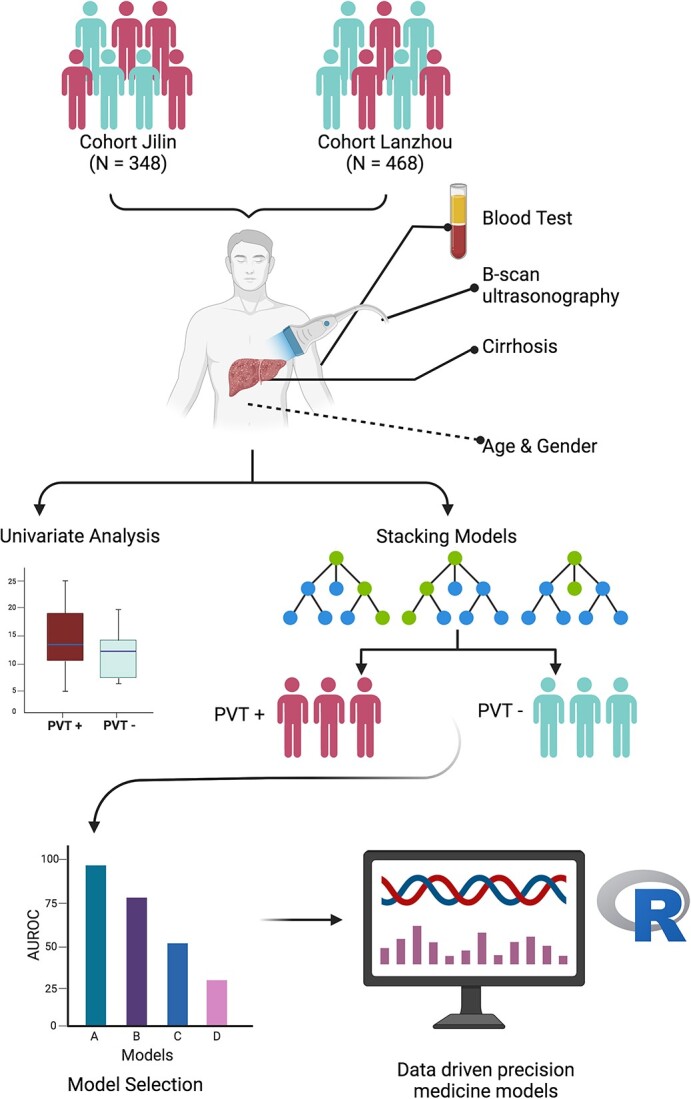
The workflow of this study.

Our patient cohort was carefully selected from two different clinical centers: the First Hospital of Lanzhou University (*n* = 468) and Jilin Hepatobiliary Hospital (*n* = 348) (Figure 1). Patients diagnosed with decompensated chronic hepatitis cirrhosis between January 2016 and December 2021 were screened again according to cirrhosis treatment guidelines published in 2019 [23]. The diagnosis of cirrhosis was based on clinical, laboratory and ultrasound evidence, and the diagnosis of PVT was based on abdominal ultrasound and liver CT scan. Exclusion criteria were hepatocellular carcinoma, extrahepatic malignancy, prior transjugular intrahepatic portosystemic shunt (TIPS) treatment, partial splenic embolization, other abdominal surgery, use of drugs known to interfere with clotting and known hemostatic disorders other than cirrhosis. None of the patients in the First Hospital of Lanzhou University had undergone splenectomy, while all the patients in Jilin Hepato-Biliary Diseases Hospital had undergone splenectomy. All these studies were reviewed by the Ethics Committee of the First Hospital of Lanzhou University.

### Data collection

To ensure a comprehensive dataset for our analysis and model development, we employed a retrospective data collection methodology. This comprehensive approach encompassed the collection of demographic information, including age and gender, as well as a wide array of clinical parameters. These parameters included the presence of portal emboli and an extensive panel of laboratory measurements. Hematological parameters such as white blood cell count (WBC, 10^9^/l), hemoglobin (HB, g/l) and platelet count (PLT, 10^9^/l) were meticulously recorded, detected by Mindray Fully Automatic Hematology Analyzer BC-6800 Plus. Albumin (ALB, g/L) and total bilirubin (TB, μmol/L) were recorded, detected by Beckman Coulter Chemistry Analyzer AU5800. Coagulation profile indicators like prothrombin time (PT, s), prothrombin activity (PTA, %), international normalized ratio (INR), fibrinogen (FIB, g/l) and activated partial thromboplastin time (APTT, s) were recorded, detected by Instrumentation Laboratory ACL TOP 750 LAZ. Radiological metrics were also systematically documented. This encompassed ascites, portal vein diameter (PVD, mm), splenic vein diameter (SVD, mm) and portal velocity (PV, cm/s), detected by Philips Doppler Ultrasonic Diagnostic Instrument EPIQ-5. Based on clinical data including ALB, TB, PT, ascites and hepatic encephalopathy judged and recorded by clinicians, the Child–Pugh score (CPS) was calculated.

### Univariate analysis

Continuous variables were presented as the means and SDs, and for skewed continuous variables, medians and interquartile ranges were used. Categorical data were expressed as number and percentage. Tests between two groups were conducted using the *t*-test, Wilcoxon test or chi-square test, as appropriate. All statistical analyses were performed using SAS Studio. All *P*-values less than 0.05 were considered statistically significant.

### The predictable models

#### The stacking model used in this study

We combined the general features, blood, B-scan ultrasonography scan and cirrhosis in the Lanzhou cohort (*N* = 468) as a training cohort. Then, we trained two machine learning models, SVM and Naïve Bayes classifier, based on the Lanzhou cohort. We analyzed the features applied in these two models using SHAP, which returned the importance of each feature. To further improve the performance, we selected the common features between the 10 most critical features adopted in these two models. These common features were fed into the QDA for training. Finally, we evaluated the performance of QDA and compared it to other machine learning methods on the Jilin cohort (*N* = 348).

#### SVM

SVM is a supervised machine learning algorithm that tries to find a hyperplane to separate two classes with the largest margin [24]. Suppose the features of a training sample *i* were ${X}_i={x}_1,{x}_2,\dots, {x}_n$, such as PT and CPS in our case. The label of the sample was ${y}_i$, where ${y}_i=1$ represents the sample with PVT and ${y}_i=-1$ denotes a non-PVT sample. Suppose the hyperplane that can separate two classes was


(1) \begin{equation*} W{X}_i+b=0 \end{equation*}

where $W={w}_1,{w}_2,\dots, {w}_n$ was a set of weights for the features, *b* was a constant. We try to find the hyperplane ${H}_0$ such that $W{X}_i+b\ge 1$ when ${y}_i=1$ and $W{X}_i+b\le -1$ when ${y}_i=-1$. Suppose ${H}_1$ and ${H}_2$ were two hyperplanes $W{X}_i+b\ge 1$ and $W{X}_i+b\le -1$. The distance between ${H}_1$ and ${H}_2$ was


(2) \begin{equation*} \frac{2\left|W{X}_i+b\right|}{{\left\Vert W\right\Vert}^2}=\frac{2}{{\left\Vert W\right\Vert}^2} \end{equation*}

The main idea of SVM was to maximize the margin between two classes, which means maximizing the distance between ${H}_1$ and ${H}_2$. That is to minimize $\frac{{\left\Vert W\right\Vert}^2}{2}$. Thus, the problem can be expressed as


(3) \begin{equation*} \min \frac{{\left\Vert W\right\Vert}^2}{2}\ s.t.{y}_i\left(W{X}_i\right)-b-1=0 \end{equation*}

which was a constrained optimization problem. By using Lagrangian, we can solve the problem that separate PVT and non-PVT subjects.

#### Naïve Bayes

The Naive Bayes method was a supervised machine learning algorithm that used for classification tasks, like text classification [25]. Suppose the features of a subject were ${X}_i={x}_1,\dots, {x}_n$, and $y$ is the label of the subject. The theorem of Naive Bayes methods is as follows:


(4) \begin{equation*} P\left(y\mid{x}_1,\dots, {x}_n\right)=\frac{P(y)P\left({x}_1,\dots, {x}_n\mid y\right)}{P\left({x}_1,\dots, {x}_n\right)} \end{equation*}

Using the naive conditional independence assumption that


(5) \begin{equation*} P\left({x}_i|y,{x}_1,\dots, {x}_{i-1},{x}_{i+1},\dots, {x}_n\right)=P\left({x}_i|y\right) \end{equation*}

Thus, the equation is simplified to


(6) \begin{equation*} P\left(y\mid{x}_1,\dots, {x}_n\right)=\frac{P(y){\prod}_{i=1}^nP\left({x}_i\mid y\right)}{P\left({x}_1,\dots, {x}_n\right)} \end{equation*}

Since $P\left({x}_1,\dots, {x}_n\right)$ is constant given the input, we can remove the denominator from this equation:


(7) \begin{equation*} P\left(y\mid{x}_1,\dots, {x}_n\right)\propto P(y){\prod}_{i=1}^nP\left({x}_i\mid y\right) \end{equation*}

So far, the discussion has yielded the independent feature model, often known as the Naive Bayes model. This model is used with a decision rule in the Naive Bayes classifier.

One typical approach is to choose the most likely hypothesis in order to reduce the likelihood of misclassification; this is known as the maximal a posteriori (MAP) decision rule. The corresponding Bayes classifier is the function that assigns a class label $\hat{y}$ as follows:


(8) \begin{equation*} \hat{\mathrm{y}}=\mathit{\arg}\kern0.5em \underset{y}{\max}\kern0.5em P(y){\prod}_{i=1}^nP\left({x}_i\mid y\right) \end{equation*}

#### SHAP

SHAP is based on Shapley values, which are a popular cooperative game theory strategy [26]. The original form of the Shapley value was used to fairly determine a player’s contribution to the final outcome of a game. Suppose we have a cooperative game where a set of players each collaborate to create some value. If we can measure the total payoff of the game, then the Shapley value reflects the marginal contribution of each participant.

If we consider our machine learning model to be a game in which individual features ‘cooperate’ to generate an output, which is a model prediction, then we may credit the prediction to each of the input features. For example, assuming teamwork is needed to finish a project. The team, *Q*, has *x* members. The total value achieved through this teamwork is *v* = *v*(*Q*). The Shapley value, ${\mathcal{g}}_{\mathrm{m}}\left(\mathrm{v}\right)$, is the fair share or payout to be given to each team member m, which is defined as


(9) \begin{equation*} {\mathcal{g}}_m(v)=\frac{1}{x}\ \sum_S\frac{\left[v\left(S\cup \left\{m\right\}\right)-v(S)\right]}{\left(\genfrac{}{}{0pt}{}{x-1}{k(S)}\right)},m=1,2,3,\dots x \end{equation*}

For a given member, *m*, the summation is over all the subsets *S*, of the team, *Q* = {1,2,3,…,*x*},that one can construct after excluding *m*. In the above formula, *k*(*S*) is the size of *S*, *v*(*S*) is the value achieved by subteam *S* and *v*(*S*∪{*m*}) is the realized value after *m* joins *S*.

Applications of Shapley values can be found in numerous areas. They can be applied to machine learning to account for feature contributions, where the features are players or team members and the model predictions are expenditures for the game or team.

#### QDA

QDA is a powerful classification method based on modeling the distribution of the data [27]. Unlike linear discriminant analysis, QDA assumes different covariances for different classes, which allows the decision boundary to be quadratic and more flexible. Assume the training data are $\left({X}_i,{y}_i\right),i=1,\dots, n$, where ${X}_i$ contains seven features, namely, PV, PT, PTA, PVD, APTT, age and CPS, ${y}_i=1$ represents the sample were PVT and ${y}_i=-1$ for non-PVT. Then, the quadratic discriminant function was


(10) \begin{align*} \delta (X)=&{X}^T{\left({\Sigma}_1-{\Sigma}_2\right)}^{-1}X+2{\left({\Sigma}_2^{-1}{\mu}_2-{\Sigma}_1^{-1}{\mu}_1\right)}^TX\nonumber \\ &+\left({\mu_1}^T{\Sigma}_1^{-1}{\mu}_1-{\mu_2}^T{\Sigma}_2^{-1}{\mu}_2\right)+\ln \left(\frac{\left|{\Sigma}_1\right|}{\left|{\Sigma}_2\right|}\right)+2\ln \left(\frac{\pi_2}{\pi_1}\right) \end{align*}

where ${\mu}_1$ and ${\mu}_2$ are the means of PVT and non-PVT classes, ${\Sigma}_1$ and ${\Sigma}_2$ are the covariance matrices of PVT and non-PVT, ${\pi}_1$ and ${\pi}_2$ are the prior probabilities of the two classes.

The classification rule is


(11) \begin{equation*} \hat{G}(X)={\mathit{\arg}\max}_k\ {\delta}_k(X) \end{equation*}

The predicted class is the class *k* that maximizes the quadratic discriminant function $\hat{G}(X)$. If ${\delta}_1(X)>{\delta}_2(X)$, the subject is predicted to be PVT. Otherwise, the subject is non-PVT.

Regularization is a common technique to improve the estimates by controlling the shrinkage of the individual class covariance matrix estimates toward the pooled estimate. The regularization hyperparameter of QDA was tuning in the training cohort using 5-fold cross validation. After the optimal regularization parameter was obtained, the QDA was trained on the entire training cohort.

#### Evaluation and comparison

In this study, we use the AUROC (area under the receiver operating characteristic curve) to evaluate the performance of our stacking model [28]. AUROC is a valuable metric for assessing the overall performance of a binary classification model, especially when dealing with imbalanced datasets or when the costs of false positives and false negatives are different. The ROC (receiver operating characteristic) curve is a graphical representation of a binary classification model’s performance across different threshold values. It depicts the true-positive rate (TPR; sensitivity) against the false-positive rate (FPR; 1-specificity) as the threshold for classifying positive instances varying. The TPR and FPR were calculated as follows:


(12) \begin{equation*} \mathrm{TPR}=\frac{\mathrm{TP}}{\mathrm{TP}+\mathrm{FN}} \end{equation*}

(13) \begin{equation*} \mathrm{FPR}=\frac{\mathrm{FP}}{\mathrm{FP}+\mathrm{TN}} \end{equation*}

where TP is the number of true-positively classified samples, FN is the number of false-negatively classified samples, FP is the number of false-positively classified samples and TN is the number of true-negatively classified samples.

We calculate the area under the ROC curve. It quantifies the model’s ability to discriminate between the positive and negative classes across all possible threshold values. The AUROC value ranges from 0 to 1, where a higher AUROC indicates better discrimination. Based on the AUROC, we compared our model with traditional machine learning methods including nearest neighbors, SVM, Gaussian process, decision tree, random forest, neural network, adaptive boosting, Naïve Bayes and LASSO.

## RESULTS

### Differential analysis of clinical features in PVT patients

We performed statistical comparisons of 13 continuous clinical parameters using either *t*-tests (for normally distributed continuous variables) or Wilcoxon tests (for skewed distribution continuous variables) as indicated in Table 1. Among these parameters, two continuous variables exhibited a significant decrease in the PVT group in both cohorts (*P*-value <0.05), i.e. HB and PTA. Conversely, three clinical variables displayed a significant increase in the PVT group. These variables encompass INR, CPS and PVD. These notable differences in clinical parameters highlight the potential for constructing predictive models based on these distinctive clinical features.

**Table 1:** Basic characteristics in the two cohorts

Variables	Lanzhou cohort			Jilin cohort		
	PVT	Non-PVT	*P*-value	PVT	Non-PVT	*P*-value
	(*N* = 221)	(*N* = 247)		(*N* = 50)	(*N* = 298)	
Gender (male)	136 (61.5%)	135 (54.7%)	0.1322	41 (82.0%)	199 (66.8%)	0.0313
Age (years)	51.6 ± 11.8	53.2 ± 11.5	0.1261	55.3 ± 8.1	53.2 ± 10.7	0.1004
WBC (10^9^/l)	3.3 ± 2.0	4.1 ± 2.2	<0.0001	4.1 ± 2.3	4.5 ± 2.3	0.3080
HB (g/l)	108.2 ± 27.5	121.0 ± 31.6	<0.0001	112.4 ± 28.7	123.1 ± 30.0	0.0194
PLT (10^9^/l)	52.0 (38.0, 69.0)	61.0 (46.0, 95.0)	<0.0001	53.0 (38.0, 92.0)	68.0 (45.0, 109.0)	0.0852
PT (s)	15.7 ± 3.2	14.9 ± 3.5	0.0129	14.7 ± 1.8	14.2 ± 3.7	0.1033
PTA (%)	63.9 ± 15.0	70.8 ± 20.7	<0.0001	69.9 ± 11.8	82.8 ± 28.3	<0.0001
INR	1.3 (1.2, 1.5)	1.2 (1.1, 1.4)	<0.0001	1.3 (1.1, 1.4)	1.1 (1.0, 1.4)	0.0038
APTT (s)	35.9 ± 6.3	36.3 ± 9.5	0.6510	38.5 ± 3.9	42.2 ± 7.6	<0.0001
FIB (g/l)	1.8 (1.6, 2.2)	2.1 (1.7, 2.7)	0.0003	2.0 (1.6, 2.5)	2.1 (1.5, 2.8)	0.9522
CPS	8.2 ± 2.0	7.4 ± 1.9	<0.0001	9.2 ± 2.2	7.3 ± 2.2	<0.0001
PVD (mm)	14.4 ± 2.8	12.8 ± 2.3	<0.0001	13.3 ± 2.9	12.5 ± 1.2	0.0470
SVD (mm)	10.4 ± 3.0	8.6 ± 3.0	<0.0001	10.2 ± 2.2	9.7 ± 1.8	0.1770
PV (cm/s)	19.9 ± 6.9	21.0 ± 5.9	0.0530	11.5 ± 1.1	11.4 ± 0.9	0.8221

Data are presented as *N* (%), mean ± SD or median (IQR).

WBC: white blood cells (10^9^/l). HB: hemoglobin (g/l). PLT: Platelet(10^9^/l). PT: prothrombin time (s). PTA: prothrombin time activity (%). INR: international normalized ratio. APTT: activated partial thromboplastin time (s). FIB: fibrinogen (g/l). CPS: Child–Pugh score. PVD: portal vein diameter (mm). SVD: splenic vein diameter (mm). PV: portal velocity (cm/s).

### Feature engineering in the Lanzhou cohort

**Figure 2:**
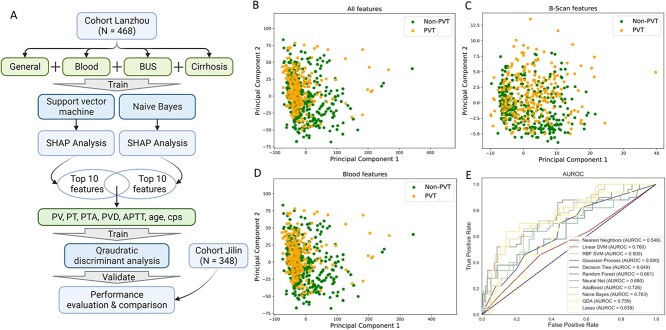
The stacking model used in this study. (**A**) The framework of the stacking model containing feature selection and machine learning model training. (**B**) The principal component visualization of all the features collected. (**C**) The principal component visualization of features from the B-scan ultrasonography scan. (**D**) The principal component visualization of features from blood. (**E**) The performance of machine learning models with all features including B-scan ultrasonography scan and blood. BUS: B-scan ultrasonography scan. SHAP: Shapley additive explanations. PV: portal velocity (cm/s). PT: prothrombin time (s). PTA: prothrombin time activity (%). PVD: portal vein diameter (mm). APTT: activated partial thromboplastin time (s). CPS: Child–Pugh score. HB: hemoglobin (g/L). SVD: splenic vein diameter (mm). WBC: white blood cell (109/L). PLT: platelet count (109/L). FIB: fibrinogen (g/L). INR: international normalized ratio.

To accurately predict PVT in chronic cirrhosis patients, we built a stacking model combining three machine learning algorithms, SVM, Naïve Bayes and QDA (Figure 2A). We used the Lanzhou cohort (*N* = 468) as the input that contains the general features, blood features, features from B-scan ultrasonography scan and cirrhosis grade. Principle component analysis shows that all these features cannot well separate PVT and non-PVT subjects (Figure 2B), neither the blood features and B-scan ultrasonography scan along (Figure 2C and D).

**Figure 3:**
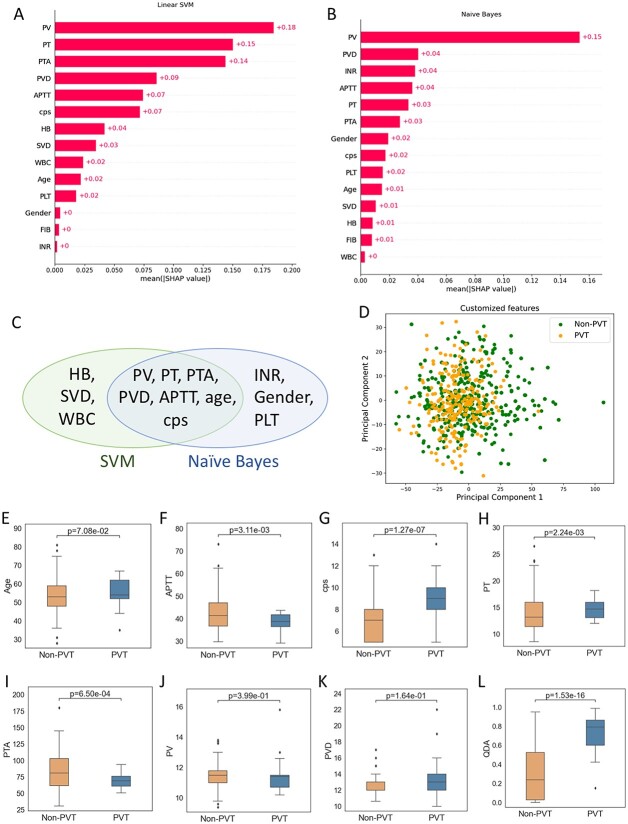
The seven common features between the top 10 features adopted in SVM and Naïve Bayes. (**A**, **B**) The SHAP value of the features in linear SVM (A) and Naïve Bayes (B) classifiers. (**C**) The Venn graph showing the top 10 features adopted in SVM and Naïve Bayes classifier based on SHAP analysis. (**D**) The principal component visualization of the common features. (**E**–**K**) The boxplot of the seven common features between PVT and non-PVT. (**L**) The score obtained by quadratic discriminant analysis between PVT and non-PVT. SHAP: SHapley Additive exPlanations. PV: portal velocity (cm/s). PT: prothrombin time (s). PTA: prothrombin time activity (%). PVD: portal vein diameter (mm). APTT: activated partial thromboplastin time (s). CPS: Child–Pugh score. HB: hemoglobin (g/l). SVD: splenic vein diameter (mm). WBC: white blood cells (10^9^/l). PLT: platelet (10^9^/l). FIB: fibrinogen (g/l). INR: international normalized ratio. SVM: support vector machine. PVT: portal vein thrombosis.

We primarily classified PVT from non-PVT using 11 machine learning algorithms training in Lanzhou cohort and testing in Jilin cohort and found that SVM and Naïve Bayes classifier outperformed other algorithms (Figure 2E). Then, we applied SHAP to explicit the features that were adopted in linear SVM (Figure 3A) and Naïve Bayes (Figure 3B). PV, PT, PTA, PVD, APTT, CPS, HB, SVD, WBC and age were the top 10 most important features in SVM. PV, PVD, INR, APTT, PT, PTA, CPS, PLT and age were the top 10 most important features calculated by Naïve Bayes.

Among the top 10 features in SVM and the top 10 features in Naïve Bayes, seven features were shared, i.e. PV, PT, PVD, PTA, APTT, age and CPS (Figure 3C). We analyzed the principal components of the seven common features (Figure 3D) and found that these features still cannot distinguish the PVT from non-PVT. We further explored the difference of the seven common features between PVT and non-PVT patients (Figure 3E–K). PTA, PV and APTT were slightly lower in the PVT patients than the non-PVT ones, whereas patients with higher PT, PVD, CPS and age were more likely to be PVT.

### Construction of PVT prediction model and validation in Jilin cohort

**Figure 4:**
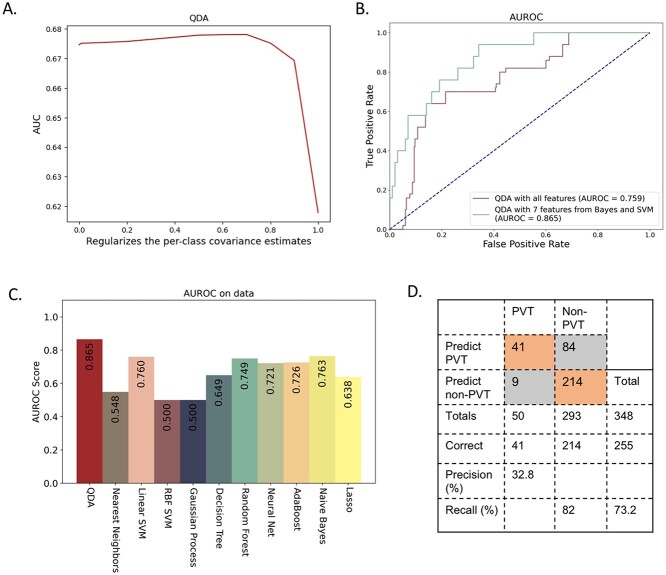
The performance of the stacking model. (**A**) The tuning of the regularization parameter in the QDA via cross-validation. (**B**) The ROC curve of the QDA with all features and seven features obtained by Naïve Bayes and SVM in the validation cohort (Jilin cohort). (**C**) The comparison among QDA and other machine learning methods based on AUROC. (**D**) The performance of our model on the validation cohort. SVM: support vector machine. AUROC: area under the receiver operating characteristic curve.

Using the seven shared features cannot well separate PVT and non-PVT due to their non-linearity with PVT. To address this problem, we applied QDA to classify PVT based on the seven common features. The QDA was trained on the Lanzhou cohort and validated on the Jilin cohort. The score obtained by QDA show significant difference between PVT and non-PVT (Figure 3L). The QDA regularization parameter was tuned using 5-fold cross validation in training cohort and was set to 0.7 (Figure 4A). The final QDA model was trained on the entire training cohort and performed well in the validation cohort (AUROC = 0.865, Figure 4B).

We compared the QDA based on the seven features (our stacking model) with other machine learning methods based on all the collected features. It outperformed nearest neighbors, SVM, Gaussian process, decision tree, random forest, neural network, adaptive boosting, Naïve Bayes and LASSO (Figure 4C). We also explored the statistics of our stacking model, which achieved an accuracy of 73.2% and a recall of 82.0% (Figure 4D). To validate the effectiveness of QDA, we compared QDA with other machine learning methods based the seven features from Naïve Bayes and SVM (Table 2). QDA was superior to other methods with AUROC of 0.865 and sensitivity of 0.820. Besides that, ablation experiments validated the effectiveness of the feature engineering of Naïve Bayes and SVM and the classifier of QDA (Table 3).

**Table 2:** The performance of quadratic discriminant analysis compared to other machine learning models based on the seven common features

Models	AUROC	Accuracy	Precision	Recall
QDA	0.865	0.733	0.328	0.820
Nearest neighbors	0.649	0.695	0.241	0.520
Linear SVM	0.762	0.695	0.254	0.580
RBF SVM	0.443	0.856	0.000	0.000
Gaussian process	0.500	0.739	0.293	0.580
Decision tree	0.681	0.649	0.223	0.580
Random forest	0.807	0.741	0.296	0.580
Neural net	0.836	0.802	0.393	0.700
AdaBoost	0.781	0.810	0.357	0.400
Naive Bayes	0.856	0.741	0.336	0.820
LASSO	0.651	0.707	0.052	0.060

LASSO: least absolute shrinkage and selection operator; QDA: quadratic discriminant analysis; SVM: support vector machine.

**Table 3:** The results of the ablation experiments

Model	AUROC
Naive Bayes	0.763
Linear SVM	0.760
(Naive Bayes and linear SVM) + logistic regression	0.786
QDA	0.759
(Naive Bayes and linear SVM) + QDA	0.865

(Naive Bayes and linear SVM) represents the common features selected by Naive Bayes and linear SVM. QDA: quadratic discriminant analysis; SVM: support vector machine.

We conducted a thorough analysis of the age factor in our model. Consequently, we attempted to eliminate the general feature, namely, age, while retaining the blood and B-scan ultrasonography scan features from the seven common features (refer to Supplemental Table S1 available online at http://bib.oxfordjournal.org/.). In the Jilin cohort, the performance of QDA improved with an AUROC of 0.870 when eliminating age in comparison to the one with all the seven features, while the precision, recall and accuracy are not decreased.

## DISCUSSION

PVT exerts a substantial impact on the quality of life and overall survival of affected individuals. Extensive research has underscored interventions initiated at earlier stages of the disease yielding significantly higher rates of successful recanalization [29, 30, 31]. Through a comprehensive analysis of clinical and laboratory data, we have meticulously crafted an advanced data-driven predictive model tailored specifically for the diagnosis of PVT in patients with chronic cirrhosis.

In our study, we harnessed data from two medical centers in China to identify a core set of clinical indicators that prove pivotal for accurate PVT prediction. This set encompasses PV, PTA, PVD, APTT, patient age and CPS. Notably, our predictive model employs a sophisticated stacking approach, amalgamating SVM, Naïve Bayes and QDA, into a comprehensive framework. It is crucial to emphasize that our model relies exclusively on objective indicators, eliminating any potential influence from subjective factors. These objective indicators encompass portal and coagulation indices, markers of liver function and age—all readily accessible in a clinical setting without incurring additional costs or effort.

Reduced PV has emerged as a pivotal risk factor for PVT in cirrhosis patients. This phenomenon contributes to the sluggish removal of clotting substances, heightened platelet–wall interactions and the occurrence of hypoxic injuries to vascular endothelial cells. Multiple studies consistently underscore that PVT incidence significantly rises when PV falls below the threshold of 15 cm/s [5, [6]. Furthermore, cirrhotic patients often exhibit enlarged portal and splenic veins, compounding the likelihood of PVT development [32, 33]. Research by Xu *et al*. introduced a predictive model for PVT, focusing on hepatitis B cirrhosis patients post-splenectomy. This model identified independent risk factors associated with PVT formation, including PVD, SVD and postoperative PLT changes [34]. Previous investigations have also suggested that factors such as splenic thickening, markedly reduced mean PV and the presence of diabetes may contribute to PVT risk [35].

The relationship between PT, PTA and PVT in cirrhosis patients is intricate. Traditionally, cirrhosis has been associated with a hypocoagulable state. However, recent evidence challenges this notion, revealing a delicate equilibrium between pro-coagulant and anticoagulant factors. Variations in this balance can lead to either hypercoagulation or hypocoagulation, and some PVT cases may even resolve spontaneously. Nonetheless, research has yielded inconsistent correlations between PVT and coagulation function in cirrhosis patients [36]. In our study, while INR, which reflects PT, showed an association with PVT, it was not integrated into the predictive model.

Our findings also emphasize the significance of the CPS in diagnosing PVT in cirrhosis. Diminished liver function is closely tied to an increased risk of PVT. The liver’s role in synthesizing coagulation and fibrinolytic factors, as well as anticoagulant substances like protein S and protein C, contributes to this intricate relationship. Liver damage disrupts these processes, affecting the clotting balance. Patients with cirrhosis may exhibit either hypocoagulation or hypercoagulation due to dynamic imbalances [37–40]. Some studies have proposed predictive models for PVT resolution based on factors like liver disease severity, thrombus characteristics and treatment timing, although the relationship between PVT and liver function scores remains inconclusive [27, 41].

The literature presents a lack of consensus regarding the impact of age on portal thrombosis. Various causes contribute to differing cirrhosis durations, and the influence of treatment on cirrhosis progression rates remains uncertain. Age has emerged as a crucial factor in PVT development, particularly in elderly patients who demonstrate heightened vulnerability. Aging introduces several mechanisms that increase the risk of PVT. Notably, oxidative stress and systemic inflammation, often associated with aging, facilitate the formation of atherosclerotic plaques, a well-established risk factor for venous thrombosis. Additionally, reduced physical activity in elderly individuals can lead to venous blood stasis, further elevating susceptibility to thrombotic events. Age-related declines in fibrinolytic activity and elevated levels of factors such as type I plasminogen activator inhibitor and platelet reactivity also play a role in augmenting the risk of venous thrombosis [42–44]. It is noteworthy that we conducted a detailed exploration of the age factor in our model. While age was initially included, its exclusion from the model—given the limited connection found in previous literature and clinical knowledge between age and portal thrombosis—did not significantly impact model efficiency. In fact, it even enhanced model performance, a noteworthy observation.

During the development of our predictive model for PVT in cirrhosis patients, we explored several associated indicators that, ultimately, did not significantly contribute to the model’s efficiency. These indicators included HB, SVD, WBC, INR, gender, D-dimer and PLT. Lower HB levels in cirrhosis patients have been linked to PVT, likely due to spleen-related issues and increased HB breakdown. However, relying solely on HB levels for diagnosis proved suboptimal, consistent with our study’s findings. Additionally, the enlargement of the SVD, potentially resulting from PVT, resulted from the obstruction of splenic vein return flow due to PVT [45]. Prior research established that a SVD exceeding 8 mm serves as the optimal diagnostic threshold for PVT [46–48]. Furthermore, elevated WBC counts often accompany infections, reflecting the intricate connection between inflammation and coagulation [49]. In cirrhosis-related PVT, systemic inflammation plays a significant role. The liver’s direct blood supply from the intestines through the portal vein connects the gut microbiome with inflammation. Even small amounts of endotoxins from gut microorganisms can trigger persistent thrombosis in cirrhosis, exacerbating clot formation [50]. Conversely, PVT development can worsen intestinal and liver ischemic damage, increasing intestinal barrier permeability [51]. Although our study showed a correlation between elevated WBC and PVT, diagnostic effectiveness was limited due to counterbalancing influences from reduced WBC due to hypersplenism [52]. D-dimer, reflecting fibrinolytic function, tends to increase during thrombosis and may also increase during hypercoagulation, infection and inflammation, but is not consistently elevated in patients with stable thrombosis without hyperfibrinolysis. The predictive value of PVT progression and prognosis may be greater than that of diagnosis [53]. Finally, advanced liver cirrhosis, often accompanied by hypersplenism, frequently leads to thrombocytopenia. Nevertheless, *in vivo* markers of platelet activation indicate that cirrhosis patients possess highly active platelets, promoting increased activation, aggregation, adhesion and release factors that elevate PVT risk [35, 54]. Consequently, platelet count was not included in our model, aligning with clinical observations.

Notably, in our study, we employed a multifaceted modeling approach, including stacking, which is also known as stacked generalization or ensemble stacking. This machine learning technique amalgamates the predictions of several base models (learners) to forge a more potent and robust model. Stacking is renowned for its ability to enhance predictive performance in comparison to individual base models. By amalgamating diverse models, stacking effectively mitigates the limitations of any single model, thereby yielding more precise and resilient predictions. Our primary stacking model harnessed the strengths of both discriminant modeling and statistical modeling by incorporating SVM and Naïve Bayes classifier. This fusion allowed us to prioritize essential features effectively. Furthermore, by integrating QDA, we achieved a quadratic boundary instead of a linear classifier. The utilization of these stacked models notably improved our ability to discern PVT accurately.

Limitations of this study should be acknowledged. Firstly, the set of clinical indicators used in the model, while informative, was not exhaustive. Additional relevant variables might exist that were not considered in this analysis, potentially impacting the accuracy and comprehensiveness of the predictive model. Secondly, differences in basic patient data between the two included centers may introduce variability in the dataset, influencing the model’s performance. The absence of follow-up data is another limitation, as it prevents the assessment of the model’s predictive capabilities over time. Lastly, the presence of selection bias in the data cannot be ignored. The patient cohorts from the two centers may not be fully representative of all cirrhosis patients, potentially affecting the model’s applicability to a broader population.

In conclusion, our study provides a robust framework for predicting PVT in chronic cirrhosis patients. Using data-driven precision medicine techniques and a model combining SVM, Naive Bayes and QDA algorithms, we focused on essential clinical indicators, including PV, PTA, PVD, APTT，CPS. This tool aids clinicians in informed decision-making regarding chronic cirrhosis and PVT. Further research and validation are needed to enhance its clinical applicability.

**Key Points**

- This study developed a data-driven predictive model for diagnosing portal vein thrombosis (PVT) in chronic cirrhosis patients, using a two cohorts consisting of 816 patients.
- Seven key clinical features were identified and assembled to conduct a stacked machine learning model, which outperformed other machine learning methods in estimating PVT risk.
- This model built in this study offers an opportunity for the early diagnosis of PVT in cirrhotic patients, which benefits clinical decision-making in managing this condition.

## Supplementary Material

Supplemental_Table-PVT-BIB_bbad478

## Data Availability

Data are available on reasonable request.

## Author Biographies

**Ying Li**, MD, is a deputy chief physician of the First Hospital of Lanzhou University. Her research interests are in is hepatology, regenerative medicine. yingli1994@126.com.

**Jing Gao**, PhD, MD, is a researcher at the Karolinska Institutet (Sweden), the University of Helsinki and Helsinki University Hospital (Finland). Her research interests include respiratory medicine,bioinformatics and global health. jing.gao@ki.se.

**Xubin Zheng**, PhD, is an assistant professor at School of Computing and Information Technology in the Great Bay University. He is also a researcher in the Great Bay Institute for Advanced Study. His research interests are in precision medicine, disease diagnosis and transcriptional regulation in cancer. xbzheng@gbu.edu.cn.

**Guole Nie** PhD student of the First School of Clinical Medicine in the Lanzhou University; His research interests are in hepatology and regenerative medicine. niegl20@lzu.edu.cn.

**Jican Qin** is a research assistant at School of Computing and Information Technology in the Great Bay University. His research interests are in precision medicine and disease diagnosis. jcqin@163.com.

**Haiping Wang**, MPH,is a researcher at the First Hospital of Lanzhou University; research direction is biostatistics. wanghp2016@hotmail.com.

**Tao He**, MD, is a clinician at Jilin Hepato-Biliary Diseases Hospital, Changchun, China, specializing in the treatment of chronic hepatitis, liver cirrhosis and liver cancer. 381418349@qq.com.

**Åsa M. Wheelock**, PhD, is an associate professor and the head of the Respiratory Medicine Unit, Department of Medicine and Centre for Molecular Medicine at the Karolinska Institutet, Stockholm, Sweden. Her research interests involve molecular sub-phenotyping of heterogeneous diagnoses of obstructive lung disease, such as COPD, asthma and post-acute sequelae of COVID-19 (PASC) using multi-omics integration and systems medicine approaches. asa.wheelock@ki.se.

**Chuan-Xing Li**, PhD, is a researcher at Karolinska Institutet (Sweden). Her research interest lies in computational precision medicine and multiomics integration. chuan-xing.li@ki.se.

**Lixin Cheng**, PhD, is a principal investigator of bioinformatics at Shenzhen People’s Hospital, Shenzhen, China. His research interests include modeling and algorithms for big data analysis in medicine and biology. easonlcheng@gmail.com.

**Xun Li**, PhD, MD, is a professor at the Department of General Surgery and Key Laboratory of Biotherapy and Regenerative Medicine of Gansu Province at the First Hospital of Lanzhou University (China). His research interests include hepatobiliary surgery, surgical endoscopy and liver transplation. lxdr21@126.com.
