# Correction to: Diagnostic Prediction of portal vein thrombosis in chronic cirrhosis patients using data-driven precision medicine model

**DOI:** 10.1093/bib/bbae252

**Published:** 2024-05-23

This is a correction to: Ying Li, Jing Gao, Xubin Zheng, Guole Nie, Jican Qin, Haiping Wang, Tao He, Åsa Wheelock, Chuan-Xing Li, Lixin Cheng, Xun Li, Diagnostic Prediction of portal vein thrombosis in chronic cirrhosis patients using data-driven precision medicine model, Briefings in Bioinformatics, Volume 25, Issue 1, January 2024, bbad478, https://doi.org/10.1093/bib/bbad478.

In the originally published version of this manuscript, the affiliations ‘The First School of Clinical Medicine, Lanzhou University, Lanzhou, China and Heart and Lung Centre, Department of Pulmonary Medicine, University of Helsinki and Helsinki University Hospital, Helsinki, Finland’ were erroneously omitted for author Jing Gao.

This error has been corrected.

