# Supplementary material for: Diagnostic Prediction of portal vein thrombosis in chronic cirrhosis patients using data-driven precision medicine model
Source: Brief Bioinform. 2024 Jan 13;25(1):bbad478. doi: 10.1093/bib/bbad478 (PMC10788706; doi:10.1093/bib/bbad478)
Supplement: Supplemental_Table-PVT-BIB_bbad478 [file supplemental_table-pvt-bib_bbad478.docx]

Supplemental Table. The performance of quadratic discriminant analysis compared to other machine learning models based on the common features excluding age.

| **Models** | **AUROC** | **Accuracy** | **Precision** | **Recall** |
| --- | --- | --- | --- | --- |
| QDA | 0.870 | 0.733 | 0.328 | 0.820 |
| Nearest Neighbors | 0.533 | 0.698 | 0.169 | 0.280 |
| Linear SVM | 0.769 | 0.741 | 0.296 | 0.580 |
| RBF SVM | 0.463 | 0.856 | 0.000 | 0.000 |
| Gaussian Process | 0.800 | 0.761 | 0.330 | 0.640 |
| Decision Tree | 0.672 | 0.641 | 0.218 | 0.580 |
| Random Forest | 0.799 | 0.810 | 0.392 | 0.580 |
| Neural Net | 0.809 | 0.718 | 0.306 | 0.760 |
| AdaBoost | 0.770 | 0.810 | 0.340 | 0.340 |
| Naive Bayes | 0.864 | 0.750 | 0.345 | 0.820 |
| Lasso | 0.651 | 0.707 | 0.052 | 0.060 |

QDA: Quadratic Discriminant Analysis; SVM: Support Vector Machine.
